# Lack of DMBT1 expression in oesophageal, gastric and colon cancers

**DOI:** 10.1038/sj.bjc.6690035

**Published:** 1999-01

**Authors:** M Mori, T Shiraishi, S Tanaka, M Yamagata, K Mafune, Y Tanaka, H Ueo, G F Barnard, K Sugimachi

**Affiliations:** Department of Surgery, Medical Institute of Bioregulation, Kyushu University, Beppu, Japan; Department of Surgery, Tokyo University School of Medicine, Tokyo, Japan; Department of Surgery, Saitama Cancer Center, Saitama, Japan; Department of Surgery, Oita Prefectural Hospital, Oita, Japan; Division of Digestive Disease and Nutrition, University of Massachusetts Medical Center, Worcester, MA USA; Department of Surgery, Kyushu University School of Medicine, Fukuoka, Japan

**Keywords:** DMBT1 gene, tumour-suppressor gene, oesophageal cancer, gastrointestinal cancer, homozygous deletion

## Abstract

Loss of sequences from human chromosome 10q has been reported in several different cancers. Recently, a second candidate tumour-suppressor gene, DMBT1, was identified in this chromosomal region. We studied the mRNA expression and homozygous deletion of this gene in human oesophageal, gastric and colon cancers. Reverse transcriptase polymerase chain reaction (RT-PCR) amplification demonstrated that 23 (53.5%) of 43 oesophageal, 5 (12.5%) of 40 gastric, and 4 (16.7%) of 24 colorectal cancer cases showed an apparent reduction in DMBT1 mRNA in tumour tissues compared with paired normal tissues. Twelve out of 15 oesophageal cancer cell lines also showed no expression. We next studied homozygous deletions within the DMBT1 gene in oesophageal cancers by using duplex PCR. Consequently, it was recognized in five (11.6%) of the primary tumours and two (13.3%) of the cell lines. These findings suggest that DMBT1 may act as a tumour-suppressor gene not only in brain tumours but also in gastrointestinal cancers, especially in oesophageal cancers. © 1999 Cancer Research Campaign


*British Journal of Cancer* (1999) **79**(2), 211–213
© 1999 Cancer Research Campaign

### Lack of DMBT1 expression in oesophageal, gastric and colon cancers

**M Mori[1], T Shiraishi[1], S Tanaka[1], M Yamagata[1], K Mafune[2], Y Tanaka[3], H Ueo[4], GF Barnard[5] and K Sugimachi[6]**

[1]Department of Surgery, Medical Institute of Bioregulation, Kyushu University, Beppu, Japan; [2]Department of Surgery, Tokyo University School of Medicine, Tokyo, Japan; [3]Department of Surgery, Saitama Cancer Center, Saitama, Japan; [4]Department of Surgery, Oita Prefectural Hospital, Oita, Japan; [5]Division of Digestive Disease and Nutrition, University of Massachusetts Medical Center, Worcester, MA, USA; and [6]Department of Surgery, Kyushu University School of Medicine, Fukuoka, Japan

**Summary** Loss of sequences from human chromosome 10q has been reported in several different cancers. Recently, a second candidate tumour-suppressor gene, DMBT1, was identified in this chromosomal region. We studied the mRNA expression and homozygous deletion of this gene in human oesophageal, gastric and colon cancers. Reverse transcriptase polymerase chain reaction (RT-PCR) amplification demonstrated that 23 (53.5%) of 43 oesophageal, 5 (12.5%) of 40 gastric, and 4 (16.7%) of 24 colorectal cancer cases showed an apparent reduction in DMBT1 mRNA in tumour tissues compared with paired normal tissues. Twelve out of 15 oesophageal cancer cell lines also showed no expression. We next studied homozygous deletions within the DMBT1 gene in oesophageal cancers by using duplex PCR. Consequently, it was recognized in five (11.6%) of the primary tumours and two (13.3%) of the cell lines. These findings suggest that DMBT1 may act as a tumour-suppressor gene not only in brain tumours but also in gastrointestinal cancers, especially in oesophageal cancers.

**Keywords:** DMBT1 gene; tumour-suppressor gene; oesophageal cancer; gastrointestinal cancer; homozygous deletion

Loss of regions of human chromosome arm 10q have been reported in various malignant tumours including malignant melanoma, endometrial cancer, pancreatic cancer, prostate cancer, small-cell lung cancer and malignant brain tumours (Gray et al, 1995; Hahn et al, 1995; Herbst et al, 1995; Peiffer et al, 1995; Albarosa et al, 1996; Petersen et al, 1997). Recently, one candidate tumour-suppressor gene was identified at chromosome 10 q, and named PTEN (phosphatase and tensin homologue deleted on chromosome 10) (Li et al, 1997). Furthermore, a second candidate tumour-suppressor gene slightly more distal on chromosome 10 q, and named DMBT1 (deleted in malignant brain tumours), was also reported (Mollenhauer et al, 1997). The lack of DMBT1 mRNA expression and the homozygous deletion of the DMBT1 gene was reported in malignant brain tumours. DMBT1 was expressed at highest levels in intestinal (and lung) tissue (Mollenhauer et al, 1997); however, there is little information on DMBT1 gene expression in human gastrointestinal cancers such as oesophageal, gastric or colorectal cancers. We, thus, studied the mRNA expression and the homozygous deletion of this gene in these common gastrointestinal cancers.

### PATIENTS AND METHODS

Paired normal and tumour total RNAs were purified from 43 oesophageal, 40 gastric and 24 colorectal cancers according to previously described methods (Li et al, 1995; Mori et al, 1995). There was no family history of cancer in these 107 cases. Of the oesophageal cancer cases, 41 were squamous cell cancers and two were small-cell cancers, histologically. All gastric and colorectal cancers were adenocarcinomas, histologically. cDNA was synthesized as described previously (Mori et al, 1995, 1998). The amplification of DMBT1 cDNA was performed in a total volume of 20 μl, which included polymerase chain reaction (PCR) buffer (10 mM tris-HCl, pH 8.3, 50 mM potassium chloride, 2 mM magnesium chloride), 200 μM dNTP, 0.5 μM each primer and 2.5 U per 100 μl of *Taq* DNA polymerase (Promega, Madison, WI, USA), and PCR conditions included an initial denaturation (94°C for 5 min), 34 cycles of amplification (94°C 1 min, 55°C 1 min, 72°C 1 min 30 s) followed by a final extension for 10 min at 72°C. We used DMBT1-specific primer sequences according to Mollenhauer et al (1997). We determined the nucleotide sequence of this PCR product and confirmed that it was identical to the expected fragment of DMBT1 cDNA. To ensure that the RNA was of sufficient purity for reverse transcriptase polymerase chain reaction (RT-PCR), a PCR assay with primers specific for the gene glyceraldehyde-3-phosphate dehydrogenase (GAPDH) cDNA was carried out in each case (Li et al, 1995; Mori et al, 1995). Aliquots of the PCR-amplified DNA were electrophoresed on 2% agarose gels containing ethidium bromide. The expression of DMBT1 and GAPDH was evaluated by computer-assisted image analysis. The tumour–normal ratio (T/N ratio) of DMBT1 expression was calculated after correction for that of GAPDH expression.

Homozygous deletions within the DMBT1 gene in the 43 primary oesophageal cancers were studied by using duplex PCR described by Mollenhauer et al (1997). The primer sequences of the target gene (g14 fragment; originally isolated as a 330-bp representational difference analysis fragment of DMBT1) and the internal control gene to DMBT1 were also described by Mollenhauer et al (1997). Aliquots of the duplex PCR-amplified DNA were electrophoresed on 2% agarose gels containing

*Received 10 February 1998*
*Revised 8 June 1998*
*Accepted 16 June 1998*

*Correspondence to:* M Mori, Department of Surgery, Medical Institute of Bioregulation, Kyushu University, 4546 Tsurumibaru, Beppu 874-0838, Japan ethidium bromide and evaluated by computer-assisted image analysis. We determined that the nucleotide sequence of the duplex PCR products was identical to the expected cDNA fragment of the target gene and the internal control gene. In each case of primary oesophageal cancer, the density ratio of g14/control in tumour tissue was divided by that in normal tissue, and a value of less than 0.3 was regarded as homozygous deletion.

The clinical significance of DMBT1 gene expression in oesophageal cancers was assessed. We compared several clinicopathological factors including histological differentiation, depth of tumour invasion in the wall, venous vessel invasion, lymphatic vessel invasion, lymph node metastasis, stage of disease, and prognosis between cases with reduced DMBT1 expression and those without reduced expression in oesophageal cancer. For statistical analysis, Fisher's exact probability test was used.

### RESULTS AND DISCUSSION

The RT-PCR results demonstrated that PCR products of the expected size were obtained from all normal mucosal tissues of the oesophagus, stomach and colorectum. Table 1 summarizes the results. An apparent reduction (T/N ratio was equal or less than 0.1), or no expression of DMBT1 mRNA, was recognized in tumour tissues compared with paired normal tissues in 23 (53.5%) out of 43 oesophageal cancer cases, 5 (12.5%) out of 40 gastric cancer cases, and 4 (16.7%) out of 24 colorectal cancer cases. Representative results of primary oesophageal cancer are shown in Figure 1A. These findings show that DMBT1 mRNA expression was remarkably suppressed in more than half of human oesophageal cancer tissues and that it was also reduced in several gastric or colorectal cancer tissues. With respect to the 15 human oesophageal cancer cell lines, the DMBT1 mRNA expression was recognized in only three cell lines (TE15, KY150 and KY36) and not recognized in the other 12 cell lines as shown in Figure 2A. In the study of Mollenhauer et al (1997), the lack of DMBT1 expression was confirmed in four out of five (80%) of the brain-tumour cell lines. The frequency of the lack of mRNA expression was the same between the brain-tumour cell lines (four out of five) and the oesophageal cancer cell lines (12 out of 15).

Mollenhauer et al (1997) showed that intragenic homozygous deletions of the DMBT1 gene were detected in 2 out of 20 medulloblastomas and 9 out of 39 glioblastoma multiforme tumours. Our study demonstrated that 5 (11.6%) of 43 primary cases of oesophageal cancer and 2 (13.3%) of the 15 cell lines showed a homozygous deletion within the DMBT1 gene (Table 1, Figures 1B and 2B). In three of these five primary cases (cases 4, 7 and 10 in Figure 1B), the expected fragment of amplified DNA was absent from tumour tissue, as shown in Figure 1B. In another two primary cases (cases 11 and 12), the expected fragment was reduced to less than 0.3 (0.10 in case 11 and 0.22 in case 12) in the tumour tissue compared with the paired normal tissue, suggesting a probable homozygous deletion of the DMBT1 gene. The weak expression might be due to a contamination by normal cells. All these five primary tumours and two cell lines (TE1 and TE14 in Figure 2B) showed no expression of DMBT1 mRNA by RT-PCR. The findings suggest that DMBT1 may act as a tumour-suppressor gene not only in brain tumours but also in oesophageal, gastric and colon cancers.

Eighteen of 23 primary oesophageal cancer cases and 10 out of 12 oesophageal cancer cell lines which either lacked or had reduced expression of DMBT1 mRNA, however, showed no intragenic homozygous deletion of this gene. Having tested g14 for deletion, this does not allow us to conclude that no other DMBT1 deletions occur in the vast majority of the tumours without expression, other mechanisms may account for the observed effect in these cases or cell lines. Frequent loss of heterozygosity at 10q23–26 in several cancers strongly suggests the presence of a

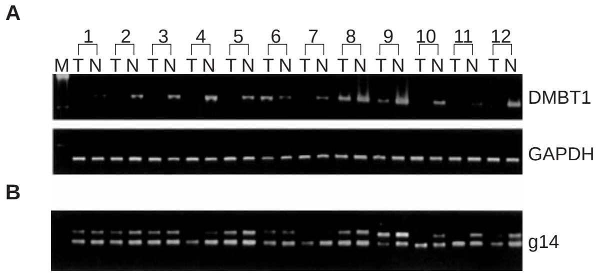

**Figure 1** (**A**) Representative results of DMBT1 expression determined by RT-PCR in 12 cases of primary oesophageal cancer. T, tumour tissue, and N, paired normal tissue, in each case. All the normal tissues examined show detectable DMBT1 expression. A remarkable reduction or lack of DMBT1 expression is evident in many oesophageal cancer tissues. The RT-PCR was performed according to a previously described method except for the number of PCR cycles (34 cycles). The size of the PCR product is 950 bp for DMBT1 and 540 bp for GAPDH (internal control). (**B**) Results of duplex PCR studying the homozygous deletion of g14 (a fragment of DMBT1) in the same cases of oesophageal cancer. The upper band (230 bp) is the fragment of g14, and the lower band (190 bp) is the fragment of the control gene. In each case of primary oesophageal cancer, the density ratio of g14/control in tumour tissue was divided by that in normal tissue and the value of less than 0.3 was regarded as homozygous deletion. The evaluated value is 1.07, 0.74, 0.91, 0, 0.77, 1.06, 0, 0.78. 1.35, 0, 0.10 and 0.22 in cases 1–12 respectively. Homozygous deletion is recognized in the tumour tissue of cases 4, 7 and 10, and also in cases 11 and 12. The faint upper band in the tumour tissue of cases 11 and 12 may be due to contamination by normal cells. These five cases showed no expression of DMBT1 mRNA in tumour tissue as shown in (**A**)

**Table 1** The lack of DMBT1 mRNA expression and homozygous deletion of the g14 fragment of the DMBT1 gene

| | Lack of DMBT1 expression (%) | Homozygous deletion of g14 (%) |
|---|---|---|
| Primary oesophageal cancers | 23/43 (53.5) | 5/43 (11.6) |
| Oesophageal cancer cell lines | 12/15 (80) | 2/15 (13.3) |
| Primary gastric cancers | 5/40 (12.5) | Not tested |
| Primary colon cancers | 4/24 (16.7) | Not tested |

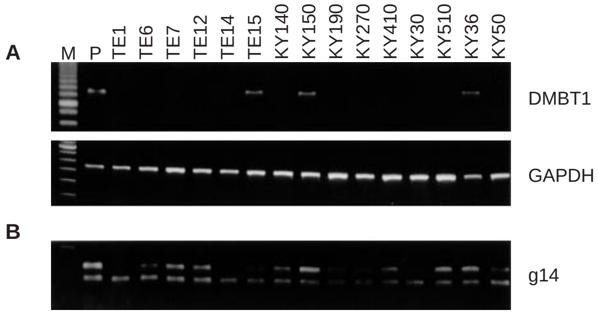

**Figure 2** (**A**) DMBT1 expression determined by RT-PCR in 15 oesophageal cancer cell lines. The expression is noticed only in TE15, KY159 and KY36. (**B**) Duplex PCR studying homozygous deletion of the g14 segment of DMBT1. Homozygous deletion is recognized in TE1 and TE14. A very faint upper band is seen in KY30, probably suggesting that one allele is lost. No expression of DMBT1 mRNA is seen in these three cell lines

*© Cancer Research Campaign 1999*

tumour-suppressor gene in this region. In fact, loss of heterozygosity of 10 q was recognized in 17% of oesophageal cancers (Aoki et al, 1994), 17% of colon cancers and 15% of gastric cancers (Kong et al, 1997). Our study demonstrated that the percentage was similar to the lack of DMBT1 gene expression in colon (17%) and gastric (13%) cancers. Kong et al (1997), however, reported that the mutation frequency of the PTEN1 gene that locates at 10q23.3 and near the DMBT1 gene was rare in colon and gastric cancers. Thus, further studies on loss of heterozygosity of DMBT1 and mutation analysis of a retained allele would be necessary to confirm that DMBT1 may be a candidate tumour-suppressor gene in these common cancers.

DMBT1 shows homology to the scavenger receptor cysteine-rich (SRCR) superfamily (Mollenhauer et al, 1997), but its precise physiological function is still unknown. We, thus, studied the significance of DMBT1 expression by comparing the cases with reduced DMBT1 expression and those without reduced expression in oesophageal cancer. There was, however, no significant difference in any of the clinicopathological factors between the two groups (Table 2). Interestingly, the case with reduced expression had a tendency to show cancer cell invasion into the vascular vessels, but this did not reach a statistically significant difference. Reduction of DMBT1 mRNA expression was recognized even in early-stage disease (stage I of the TNM classification), suggesting that it could be an early event in the development of oesophageal cancer. This is in contrast to malignant brain tumours in which allelic losses were noted in none of ten low-grade astrocytomas (Mollenhauer et al, 1997). Further detailed search for the biological function of this gene is necessary to understand its function as a postulated tumour-suppressor gene. This will encourage an understanding of the molecular mechanism of oesophageal cancer, one that has a poor prognosis.

**Table 2** Expression of DMBT1 mRNA and oesophageal cancers

| | Expression of DMBT1 mRNA | | |
|---|---|---|---|
| | T/N>0.1 (*n*=20) | T/N≤0.1 (*n*=23) | *P*-value |
| Histology | | | ns |
| Well scc | 6 | 5 | |
| Moderately scc | 12 | 11 | |
| Poorly scc | 2 | 6 | |
| Small-cell cancer | 1 | 1 | |
| Depth of tumour invasion | | | ns |
| Within the wall | 6 | 4 | |
| Adventita | 14 | 16 | |
| Invading the adjacent organ | 0 | 3 | |
| Venous vessel invasion | | | ns |
| Absent | 9 | 2 | |
| Present | 11 | 21 | |
| Lymph vessel invasion | | | ns |
| Absent | 3 | 2 | |
| Present | 17 | 21 | |
| Lymph node metastasis | | | ns |
| Absent | 4 | 3 | |
| Present | 16 | 20 | |
| Stage | | | ns |
| 1 | 2 | 1 | |
| 2 | 1 | 0 | |
| 3 | 11 | 11 | |
| 4 | 6 | 11 | |

scc, squamous cell cancer; ns, not significantly different.

### ACKNOWLEDGEMENT

This study was supported in part by a grant from the Ministry of Education, Science and Culture of Japan.

### REFERENCES

Albarosa R, Colombo BM, Roz L, Magnani I, Pollo B, Cirenei N, Giani C, Conti AM, DiDonato S and Finocchiaro G (1996) Deletion mapping of gliomas suggests the presence of two small regions for candidate tumor-suppressor genes in a 17-cM interval on chromosome 10q. *Am J Hum Genet* **58**: 1260–1267

Aoki T, Mori T, Du XO, Nishihira T, Matsubara T and Nakamura Y (1994) Allelotype study of esophageal carcinoma. *Genes Chroms Cancer* **10**: 177–182

Gray IC, Phillips SM, Lee SJ, Neoptolemos JP, Weissenbach J and Spurr NK (1995) Loss of the chromosomal region 10q23–25 in prostate cancer. *Cancer Res* **55**: 4800–4803

Hahn SA, Seymour AB, Hoque ATMS, Schutte M, da Costa LT, Redston MS, Caldas C, Weinstein CL, Fischer A, Yeo CJ, Hruban RH and Kern SE (1995) Allelotype of pancreatic adenocarcinoma using xenograft enrichment. *Cancer Res* **55**: 4670–4675

Herbst R, Weiss J, Ehnis A, Cavenee WK and Arden KC (1995) Loss of heterozygosity for 10q22–10qter in malignant melanoma progression. *Cancer Res* **54**: 3111–3114

Kong D, Suzuki A, Zou TT, Sakurada A, Kemp LW, Wakatsuki S, Yokoyama T, Yamakawa H, Furukawa T, Sato S, Yin J, Wang S, Abrahan JM, Souza RF, Smolinski KN, Meltzer SJ and Horii A (1997) PTEN1 is frequently mutated in primary endometrial carcinomas. *Nature Genet* **17**: 143–144

Li J, Mori M, Yang Y, Inoue H, Mimori K, Shibuta K, Nakashima H, Mafune K, Shimada Y, Barnard GF, Sugimachi K and Akiyoshi T (1995) Multiple tumor suppressor 1 gene and esophageal carcinoma. *Int J Cancer* **7**: 257–260

Li J, Yen C, Liaw D, Podsypanina K, Bose S, Wang SI, Puc J, Miliaresis C, Rodgers L, McCombie R, Bigner SH, Giovanella BC, Ittmann M, Tycko B, Hibshoosh H, Wigler MH and Parsons R (1997) PTEN, a putative protein tyrosine phosphatase gene mutated in human brain, breast, and prostate cancer. *Science* **275**: 1943–1947

Mollenhauer J, Wiemann S, Scheurlen W, Korn B, Hayashi Y, Wilgenbus K, von Deimling A and Poustka A (1997) DMBT1, a new member of the SRCR superfamily, on chromosome 10q25.3–26.1 is deleted in malignant brain tumors. *Nature Genet* **17**: 32–39

Mori M, Mimori K, Inoue H, Barnard GF, Tsuji K, Nanbara S, Ueo H and Akiyoshi T (1995) Detection of cancer micrometastases in lymph nodes by reverse transcriptase-polymerase chain reaction. *Cancer Res* **55**: 3417–3420

Mori M, Mimori K, Ueo H, Tsuji K, Shiraishi T, Barnard GF, Sugimachi K and Akiyoshi T (1998) Clinical significance of molecular detection of carcinoma cells in lymph nodes and peripheral blood by reverse transcription-polymerase chain reaction in patients with gastrointestinal or breast carcinomas. *J Clin Oncol* **16**: 128–132

Peiffer SL, Herzog TJ, Tribune DJ, Mutch DG, Gersell DJ and Goodfellow PJ (1995) Allelic loss of sequences from the long arm of chromosome 10 and replication errors in endometrial cancers. *Cancer Res* **54**: 1922–1926

Petersen I, Langreck H, Wolf G, Schwendel A, Psille R, Vogt P, Reichel MB, Ried T and Dietel M (1997) Small-cell lung cancer is characterized by a high incidence of deletions on chromosomes 3p, 4q, 5q, 10q, 13q, and 17p. *Br J Cancer* **75**: 79–86

© Cancer Research Campaign 1999
